# White Fibrous Papulosis of the Neck in a 70-Year-Old Female: A Case Report

**DOI:** 10.7759/cureus.51957

**Published:** 2024-01-09

**Authors:** Maryam Alzaabi, Rawan Almutairi, Amal Alrushood, Humoud Al-Sabah

**Affiliations:** 1 Dermatology, As'ad K. Al-Hamad Dermatological Center, Kuwait, KWT; 2 Dermatopathology, As'ad K. Al-Hamad Dermatological Center, Kuwait, KWT

**Keywords:** dermatopathology, skin lesion biopsy, rare skin disease, fibroelastic papulosis of the neck, collagen thickening, decrease in elastic fibers, white fibrous papulosis of the neck

## Abstract

White fibrous papulosis of the neck (WFPN) manifests through the presence of numerous solid, persistent, and asymptomatic yellowish-white papules, displaying a distinctive asymmetrical distribution primarily localized on the neck and antecubital fossa. This case report describes the clinical presentation of a 70-year-old female diagnosed with WFPN, highlighting the significant finding of collagen fiber thickening upon histopathological analysis. Despite its predilection for specific anatomical sites, the elusive pathogenesis of WFPN adds diagnostic complexity, emphasizing the need for further research in this unique condition that generally follows a benign course.

## Introduction

White fibrous papulosis of the neck (WFPN) represents an uncommon benign disease characterized by the symmetrical distribution of numerous tiny white pale papules that are asymptomatic and located on the neck. WFPN was first identified in Japan in 1985 [1]. While rarely observed in areas such as the back, thorax, or antecubital fossa, WFPN primarily manifests on the neck. The demographic trend indicates a predilection for elderly women, although the etiology remains elusive, devoid of known associated risk factors. The actual prevalence of this condition is understated [2]. Here, we present a case of WFPN in a 70-year-old woman who exhibited whitish, non-pruritic papules on the sides of her neck and antecubital fossa for a duration of two years.

## Case presentation

A 70-year-old previously healthy woman presented with asymptomatic, non-pruritic yellowish papules on the side of the neck and antecubital fossa bilaterally, which started two years prior to the presentation. The lesions did not progress, nor did they cause any itchiness or physical discomfort. The patient denied a history of prolonged sun exposure, rubbing or scratching of affected areas, or cutaneous growth lesions in her body before or during pregnancy or weight gain. She was healthy, non-diabetic, and non-hypertensive. No fever or other systemic symptoms were noted. Family and medical histories were insignificant.

**Figure 1:**
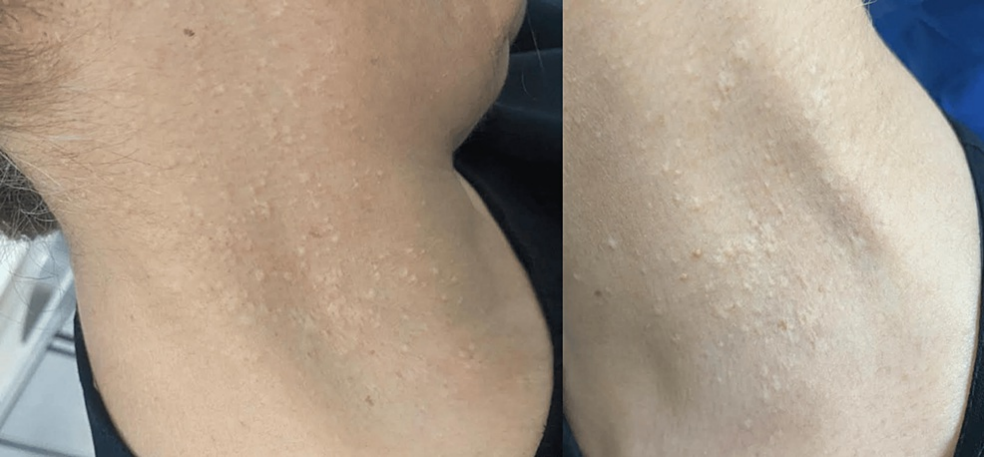
Multiple polygonal flat-topped, yellowish-white papules are scattered on both sides of the neck

**Figure 2:**
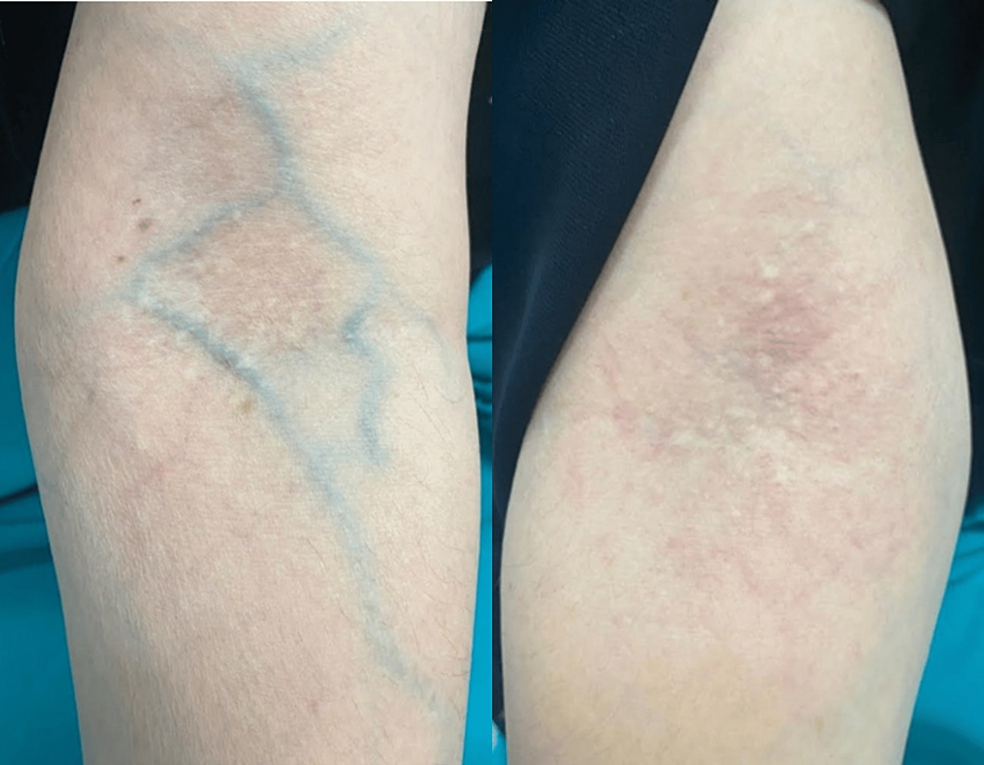
The same lesion type is also present on both antecubital fossae

Dermatological examination revealed multiple polygonal flat-topped yellowish-white papules scattered on both sides of the neck, giving a pattern of plucked chicken skin (Figure 1). Upon examination of her upper limbs, the same lesions were scattered bilaterally on both antecubital fossae (Figure 2). Skin lesions were confined to these areas. Physical examination was completed with fundoscopy and cardiovascular assessment, both of which were unremarkable.

**Figure 3:**
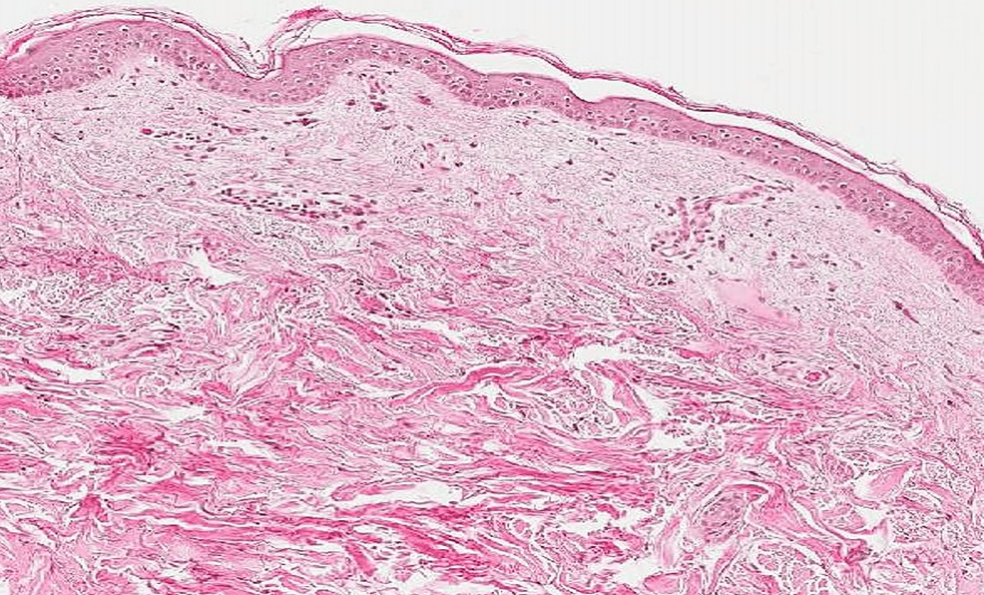
Hematoxylin and eosin stain revealing folded epidermis with hyperkeratosis and orthokeratosis with thickening of collagen fibers in the superficial and mid dermis .

**Figure 4:**
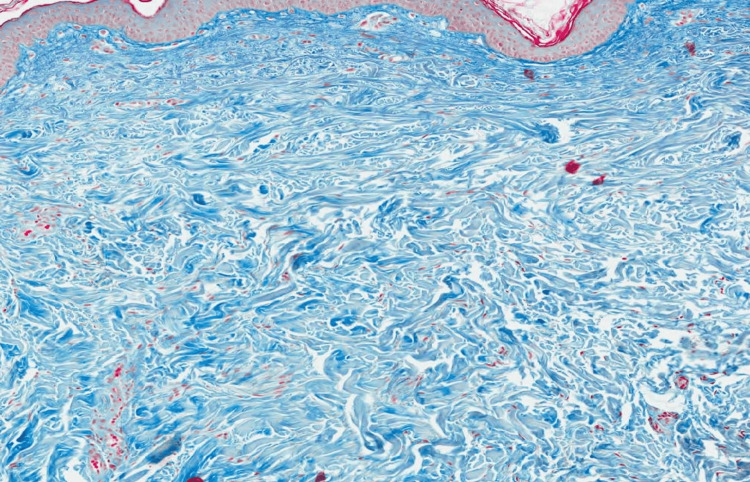
Masson’s trichome stain showing collagen fibers haphazardly arranged and thickened in the papillary dermis

**Figure 5:**
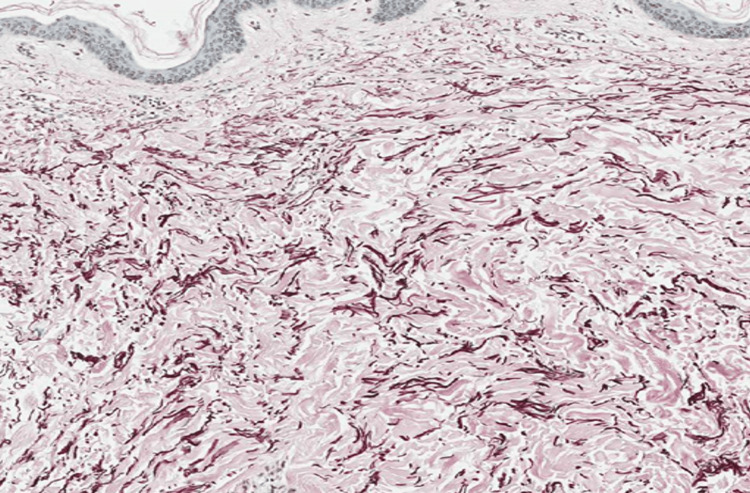
Elastic Van Gieson stain revealing normal elastic fibers in a normal pattern and quantity

Histological evaluation revealed a folded epidermis with hyperkeratosis and orthokeratosis with thickening of collagen fibers in the superficial and medium dermis on hematoxylin and eosin staining (Figure 3). Masson’s trichrome staining revealed haphazardly arranged and thickened collagen fibers in the papillary dermis (Figure 4). While assessing the pattern of fibers, elastic Van Gieson staining revealed normal elastic fibers in a normal pattern and quantity (Figure 5).

Based on the clinical and histopathological findings, the diagnosis of WFPN was reached. The patient was then reassured about her condition as it was found to be benign, and she received education regarding its nature. No specific treatment was required, and the patient continued to undergo observation with no changes in the lesions during follow-up examinations at two months.

## Discussion

Shimizu and Nishikawa were the first to identify white fibrous papulosis of the neck in 1985 [1]. This uncommon condition tends to affect elderly people (fifth to ninth decades), with an average age of > 60 years. WFPN belongs to the broader category of fibroelastic papulosis of the neck, which has two subcategories: pseudoxanthoma elasticum-like papillary dermal elastolysis (PXE-PDE) and WFPN. The causes of this disease are not completely understood, but aging and environmental factors such as exposure to sunlight play a role in its pathogenesis [2,3]. The relative uncommonness of this condition in the field of clinicopathology has led to an underestimation of its prevalence in the medical literature.

WFPN is frequently misdiagnosed as PXE or PXE-PDE because its clinical and histological characteristics are not precisely defined [4]. WFPN is diagnosed based on clinical presentation and histopathological analysis. The back of the neck is the most common location of papules, which are typically tiny, whitish, smooth, and clearly demarcated. To confirm the clinical diagnosis of WFPN, a biopsy is performed. It is characterized histologically by the presence of fibroelastolytic alterations in the papillary dermis, with a decrease or absence of elastic fibers [5-7]. Given that this condition impacts the neck, it is also imperative to differentiate WFPN as a benign ailment characterized by the absence of lymph node pathology [8].

These clinical features suggest the following differential diagnoses: PXE-PDE, PXE, acrochordons, fibrofolliculoma, trichodiscomas, Buschke-Ollendorff syndrome, and cutis rhomboidalis nuchae (Table 1) [2,4,5,9-13]. PXE papules resemble WFPN owing to their white, discrete appearance, which can occasionally merge, creating a "cobblestone" or "chicken skin" texture [14]. These lesions can be found not only in the neck, but also in areas such as the axilla, abdomen, groins, perineum, and thighs. Unlike WFPN, the papules in PXE exhibit distinctive histopathological features, including collagen fiber splitting and the presence of basophilic elastic fibers due to calcium deposition. In addition, patients with PXE have ocular abnormalities such as angioid streaks or maculopathy. Histologically, WFPN displays normal elastic fibers, whereas, in PXE-PDE, the elastic fibers are significantly reduced or absent in the papillary dermis [2,4].

**Table 1:** Differential diagnosis of white fibrous papulosis of the neck

Disease	Age onset	Clinical features	Histopathology
White fibrous papulosis of the neck	Elderly individuals	Neck: several round, white, asymptomatic papules around 2-3 mm in diameter.	Elastic fibers: normal to slightly decreased
pseudoxanthoma elasticum-like papillary dermal elastosis	Postmenapausal and elderly women	Soft, yellow papules with a tendency to coalesce into cobblestone plaques on the neck, simulating pseudoxanthoma elasticum	Marked decrease to absence of elastic fibers in the papillary dermis. No calcification or fragmentation seen
Pseudoxanthoma elasticum	Early childhood but rarely starts in old age	Skin signs: yellow cobblestone lesions on areas of bending like neck, axilla, abdomen, groin, perineum, and thighs Ophthalmological manifestations: angioid stretch marks, peau d’orange, maculopathy. Cardiological manifestations: intermittent claudication, coronary artery disease, arterial hypertension, angina, myocardial infarction, congestive heart failure, restrictive cardiomyopathy, and valvulopathies.	Elastic fibres appear basophilic due to calcium deposition Fragmentation and clumping are seen in the reticular dermis. Collagen fibers are split
Acrochordons	More frequent with increasing age, during second-trimester pregnancy, obese, diabetic, birth Hogg dube syndrome	Lesions are located in sites of friction, axillary, cervical, inframammary, and inguinal regions. Asymptomatic until there is a trauma to the lesions. Appear as a red or black lesion when there is torsion of their peduncle. Associated with increased risk of DM and HTN	Lesions consist mainly of fibers of collagen, fat, and other types of tissues such as blood, blood vessels, mast cells, Langerhans cells, and dermis.
Fibrofolliculoma and trichodiscomas		Papules of 2-4 mm in the shape of a meat-colored dome. Appear on the face (chin, nose, cheeks, ears, and eyebrows). Both lesions are asymptomatic and indistinguishable from each other on visual examination.	fibrofolliculoma: Dilated central follicular infundibulum, epithelial strands of basaloid cells emanating from the infundibulum of the hair follicle. Trichodiscoma: Proliferation of connective tissue and fibrous stroma, located near a hair follicle.
Buschke-Ollendorff syndrome	Before puberty	Linear cortical hyperostosis is seen in long bones in an x-ray. Connective tissue nevus is seen as papules, plaques, and yellowing or skin-colored nodules on thighs and buttocks.	Histology of connective tissue nevus: Increased elastic fibers or collagen fibers.
Cutis rhomboidalis nuchae	Elderly with fair skin	Diffuse thickening on the back of the neck with yellowing of the skin, and formation of deep grooves which results in a typical irregular rhomboidal pattern.	Thickening of the epidermis and abnormalities in the composition of the dermis.
Mid-dermal elastolysis	Young and middle-aged women	Type I patches of well-circumscribed fine wrinkles involving the trunk and proximal extremities Type II perifollicular papular protrusions Type III persistent reticular erythema and wrinkling	Selective loss of elastic fibers in the mid-dermis associated with mild lymphocytosis of elastic fibers by macrophages

Acrochordons were ruled out because the papules lack peduncles and are unrelated to pregnancy, weight gain, diabetes, or hypertension. Histologically, acrochordons consist of collagen fibers, adipose tissue, blood vessels, mast cells, and Langerhans cells in the dermis [11]. In contrast, fibrofolliculomas and trichodiscomas manifest on the face as asymptomatic, dome-shaped papules ranging in size from 2 mm to 4 mm. Histology reveals that in fibrofolliculoma, the central follicular infundibulum of the hair follicle is dilated and basaloid epithelial filaments protrude from it. The histology of trichodiscoma reveals the proliferation of a fibrous stoma and connective tissue close to a hair follicle [12]. Cutis rhomboidalis nuchae predominantly affects elderly individuals with fair skin and a history of sun exposure. This causes diffuse skin thickening, yellowing, and deep fissures on the back of the neck. Histologically, this condition is characterized by increased epidermal thickness and an atypical dermis composition [13]. In contrast to Frey's syndrome, individuals with WFPN exhibit an absence of gustatory sweating or flushing in the cheeks, temple, and post-auricular area. While WFPN predominantly affects the elderly population with an indeterminate etiology, Frey's syndrome arises as a consequence of postoperative complications related to salivary gland surgery, neck dissection, facelift procedures, and trauma impacting the parasympathetic innervation to the glands [15].

Due to the absence of bacterial pathology in WFPN, antimicrobial treatment is not included in the conservative management [16,17]. As this condition is benign and only causes cosmetic concerns for some people, only a few patients tend to seek treatment. Although there is limited information regarding WFPN treatment, a case report described the use of laser therapy, such as fractionated 1550 nm erbium glass laser, which resulted in papule clearance without adverse effects [18]. Co2 laser application also showed an excellent cosmetic resolution with improvement in itching [19]. Further research is required to determine the most effective treatment option for WFPN.

## Conclusions

WFPN is a benign skin lesion belonging to the category of fibroelastic papulosis of the neck. A 70 year old female presented with multiple discrete papular lesions in her neck and antecubital fossa. These papules were asymptomatic, pale to skin-colored, non-follicular, and firm in texture. They are predominantly found in the neck and are commonly observed in the elderly. Skin biopsy provides valuable histological insights, further aiding in the diagnosis and confirmation of the benign nature of the lesions. This case highlights the importance of thorough dermatological evaluation for accurate diagnosis of rare skin conditions.
